# Supplementary material for: Sorafenib-Regorafenib Sequential Therapy in Japanese Patients with Unresectable Hepatocellular Carcinoma—Relative Dose Intensity and Post-Regorafenib Therapies in Real World Practice
Source: Cancers (Basel). 2019 Oct 9;11(10):1517. doi: 10.3390/cancers11101517 (PMC6826625; doi:10.3390/cancers11101517)
Supplement: Supplementary file 1 [file cancers-11-01517-s001.pdf]

cancers

MDPI

Article

Sorafenib-Regorafenib Sequential Therapy in Japanese Patients with Unresectable Hepatocellular Carcinoma—Relative Dose Intensity and Post-Regorafenib Therapies in Real World Practice

Wan Wang [1,†], Kaoru Tsuchiya [1,†], Masayuki Kurosaki [1], Yutaka Yasui [1], Kento Inada [1,2] and Sakura Kirino [1,2], Koji Yamashita [1,3], Shuhei Sekiguchi [1,2], Yuka Hayakawa [1,2], Leona Osawa [1,3], Mao Okada[1], Mayu Higuchi [1,3], Kenta Takaura [1], Chiaki Maeyashiki [1,2], Shun Kaneko [1,2], Nobuharu Tamaki [1,3], Hiroyuki Nakanishi [1], Jun Itakura [1], Yuka Takahashi [1], Yasuhiro Asahina [2], Nobuyuki Enomoto [3], Namiki Izumi [1,*]

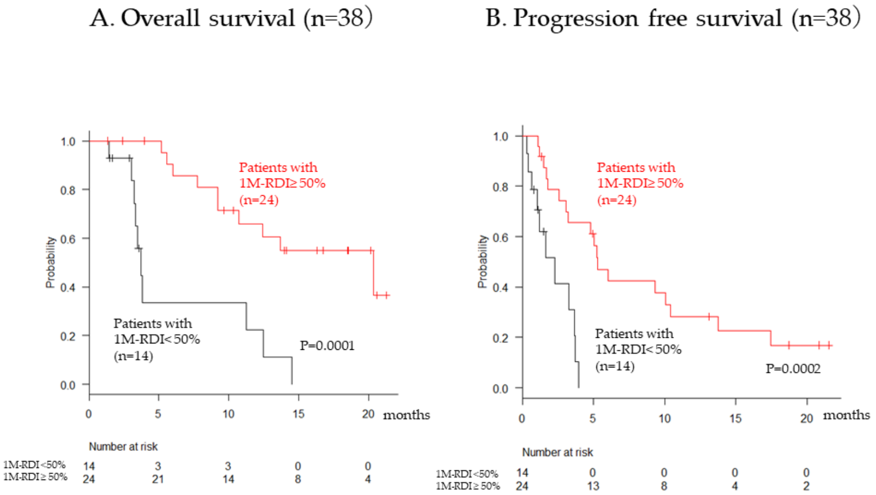

Supplementary Figure S1. Comparison between patients with 1M-RDI≥50% and <50% in overall survival (A) and progression-free survival (B).
